# Impaired expression of *BCAT1* relates to muscle atrophy of mouse model of sarcopenia

**DOI:** 10.1186/s12891-022-05332-7

**Published:** 2022-05-13

**Authors:** Hui Ouyang, Xuguang Gao, Jun Zhang

**Affiliations:** Department of Neuromedicine, Peking University People’s Hospital, 11 Xizhimen South Street, Xicheng District, Beijing, 100044 China

**Keywords:** Sarcopenia, BCAT1, mTORC1, Muscle, Muscle atrophy

## Abstract

**Background:**

The underlying mechanism of muscle atrophy in sarcopenia is still not fully understood; branched chain aminotransferase 1(*BCAT1*) isocitrate dehydrogenase-1 encodes an evolutionarily conserved cytoplasmic aminotransferase for glutamate and branched-chain amino acids (BCAAs), thus constituting a regulatory component of cytoplasmic amino and keto acid metabolism. In human gliomas carrying wild-type isocitrate dehydrogenase-1, *BCAT1* promotes cell proliferation through amino acid catabolism. Hence, the goals of this study were to unravel the potential role of *BCAT1* expression in muscle atrophy and to explore the mechanisms underlying this process.

**Methods:**

We first measured *Bcat1* expression by RT-qPCR and western blotting in murine and cellular models of muscle atrophy. To understand how the *Bcat1*-driven changes sustained muscle cell growth, we analyzed reactive oxygen species (ROS) levels and activation of the mTORC1/S6K1 pathway in muscle cells. Furthermore, we performed Cell Counting Kit-8(CCK8) assays and fluorescence staining to evaluate growth rate of cells and ROS levels. Finally, we verified that depletion of *Bcat1* impairs the growth rate of muscle cells and increases ROS levels, indicating that muscle atrophy resulted from the downregulation of the mTORC1/S6K1 pathway. Data were analyzed by two-tailed unpaired Student’s *t*-test or Mann-Whitney U test for two groups to determine statistical significance. Statistical analyses were performed using GraphPad Prism version 6.0 and SPSS 16.0 software.

**Results:**

*Bcat1* expression level in skeletal muscles was lower in murine and cellular models of sarcopenia than in the control groups. *Bcat1* knockdown not only suppressed the growth of muscle cells but also increased the production of ROS. Impaired cell growth and increased ROS production was rescued by co-introduction of an shRNA-resistant *Bcat1* cDNA or addition of the mTORC1 stimulator MYH1485. Muscle cells with *Bcat1* knockdown featured lower mTORC1 and S6K1 phosphorylation (pS6K1) than NT muscle cells. Addition of either shRNA-resistant *Bcat1* cDNA or MYH1485 rescued the suppression of cell growth, increase in ROS production, and decrease in pS6K1.

**Conclusions:**

The branched chain amino acids catabolic enzyme BCAT1 is essential for the growth of muscle cells. *BCAT1* expression contributes to sustained growth of muscle cells by activating mTOR signaling and reducing ROS production.

**Supplementary Information:**

The online version contains supplementary material available at 10.1186/s12891-022-05332-7.

## Background

Sarcopenia, the loss of skeletal muscle mass and function, is a debilitating consequence of cachexia, neuromuscular diseases, and diseases related to nerve injury. This loss of muscle function reduces the ability to recover from exercise. Therefore, patients with sarcopenia are at increased risk of physical weakness and death. Applying the European Working Group on Sarcopenia (EWGSOP) definition of sarcopenia in a community-dwelling population (the UK-based Hertfordshire Cohort Study), researchers reported that sarcopenia was present in 4.6% of men and 7.9% of women at a mean age of 67 years [1].

The etiologies of sarcopenia include aging, drug abuse, lack of activity, disease, and malnutrition, among others, many of which often coexist. However, the underlying mechanisms of muscle atrophy in sarcopenia are not fully understood [2].

Branched-chain amino acids (BCAAs) are essential amino acids that play an important regulatory role in nutrient signaling, body nitrogen metabolism, and protein synthesis [3]. The first enzymatic step is the reversible transamination catalyzed by branched chain amino acid transaminase (BCAT) isozymes (mitochondrial BCATm and cytosolic BCATc) [4]. The second step is the oxidative decarboxylation of branched-chain α-keto acids (BCKAs) to their respective branched chain acyl-CoA derivatives, which is coupled with the formation of nicotinamide adenine dinucleotide (NADH) [5].

Branched chain amino acid transaminase 1(BCAT1) is an enzyme that initiates the catabolism of BCAAs. *BCAT1* encodes an evolutionarily conserved cytoplasmic transaminase that acts on glutamate and BCAAs and thus constitutes a regulatory component of cytoplasmic amino and keto acid metabolism. Although skeletal muscle represents the most important site for BCAA transamination, the role of *BCAT1* in human sarcopenia remains unknown. *BCAT1* is well studied in immune cells like macrophages, and *BCAT1* influence cell proliferation through mammalian target of rapamycin (mTOR) signaling [6]. However, whether *BCAT1* affects the muscle cells though mTOR pathway have not been studied yet.

Low expression levels of BCATm and BCKDC were found to be associated with elevated plasma BCAAs [7], which may affect the mTOR pathway, more specifically, mammalian target of rapamycin complex 1 (mTORC1), in peripheral tissues and influence protein synthesis [8], it is a conserved serine/threonine kinase that plays a key role in cell growth and metabolism [9, 10]. The researches above suggested that *BCAT1* may influence protein synthesis and cell growth through mTOR pathway. The typical characteristic of mTOR activity is the decrease in phosphorylation of the downstream substrate ribosomal S6 kinase 1(S6K1). Activation of the mTOR pathway (through mTORC1) can stimulate protein synthesis [11]. Furthermore, mTOR activity regulates protein synthesis by integrating and transforming various extracellular cues from growth factors and nutrients, as well as those related to cellular energy status and environmental pressure [12]. Among the two different intracellular mTOR complexes (mTORC1 and mTORC2), mTORC1 contains regulatory proteins related to mTOR, and is sensitive to rapamycin. Reportedly, mTORC1 is the most important regulator of protein synthesis [13], as it phosphorylates its downstream effectors S6K1 and 4E-binding protein (4E-BP) in a rapamycin-sensitive manner [14, 15]. Additionally, mTORC1 regulates protein translation, cell proliferation, apoptosis, and autophagy. The mTORC1/S6K1 axis transmits and integrates important signals that are essential for biological processes involving nutrients, growth factors, and energy metabolites.

Previous studies have shown that mTORC1 activation was associated with muscle hypertrophy [16]. In addition, muscle-specific *raptor* gene knockout mice showed severe muscle atrophy and weight loss, leading to early death [17].

Therefore, the aims of this study were to investigate the potential role of *BCAT1* expression in muscle atrophy, and to further explore the underlying mechanism. We first measured the expression of *Bcat1* in murine and cellular models of muscle atrophy. To understand how *Bcat1*-driven changes affected muscle cell growth, we analyzed reactive oxygen species (ROS) levels in muscle cells and activation of the mTORC1/S6K1 pathway. Finally, we validated our hypothesis that *Bcat1* depletion could induce muscle atrophy by downregulating the mTORC1/S6K1 pathway and increasing ROS levels.

## Methods

### Mice

Ten Male C57BL/6 mice were randomly assigned to the following two groups of 5 mice each: (1) the control group, (2) dexamethasone (DEX)-induced sarcopenia group. Dead or ill mice that could not cooperate were removed from the experiment, and no mice were removed.

The mice were obtained from the Health Science Center of Peking University. In this study, the average age of the animals was 10 weeks. All mice were sacrificed by cervical dislocation after the experiment. Fresh muscle samples were stored at − 80 °C. All animal experiments were approved by the Animal Care and Use Committee of Peking University People’s Hospital.

### Generation of a mouse model of sarcopenia

The control group was intraperitoneally injected with sterile normal saline over 10 days, and the sarcopenia group was intraperitoneally injected with 25 mg/kg/day of water-soluble DEX (Sigma Aldrich) for 10 days [18]. The animals were euthanized by cervical dislocation on day 11. Body weights were measured, and total muscle mass, tibial anterior muscles, and gastrocnemius muscles were removed, weighed, and quickly frozen for analysis of mRNA and protein levels.

### Cell culture

Murine C2C12 myoblasts were cultured as recommended by the supplier (Cell Resource Bank of the Chinese Academy of Sciences). In short, C2C12 myoblasts were maintained in DMEM supplemented with 10% FBS, 100 U/mL penicillin, and 100 U/mL streptomycin. For routine differentiation, the cells were grown to approximately 80% confluence.

### Immunofluorescence staining

C2C12 cells were cultured in multiwells, treated with DEX (1 μM) or sterile saline for 24 h, fixed with 4% paraformaldehyde at 4 °C, washed three times with phosphate-buffered saline (PBS), permeabilized with 0.3% Triton X-100, washed three times with PBS, blocked with 10% goat serum in PBS, and incubated with primary antibody diluted in PBS (1:200) at 4 °C. After three sequential washes with PBS, the cells were incubated with fluorescently labeled donkey anti-rabbit IgG (H + L) as secondary antibody (594) diluted in PBS (1:300) at 4 °C, and washed three times with PBS afterwards. The nuclei were counterstained with DAPI and subsequently washed with PBS. Digital images of fluorescently labeled cells were obtained using a fluorescence microscope (Olympus IX51, Japan).

### SDS-PAGE and western blot analysis

The cell samples were directly dissolved in cryolysis buffer [RIPA buffer (Beyotime, China), PMSF (Amresco, China), cocktail (Shanghai Yuanye, China), Triton X-100 (China Amresco), and NaVO3] for 15 min. The lysates were clarified by centrifugation (12,000×*g,* 15 min at 4 °C). The supernatant was collected and stored at − 80 °C. Protein concentration was determined using the Beyond Time™ BCA Test Kit (Shanghai Beiotim Biotechnology Company, China). For western blotting, SDS-PAGE was used to separate the same amount of protein per sample, which was then transferred to a PVDF membrane (Millipore, USA). Afterwards, the membrane was incubated with 5% skimmed milk in Tris-buffered saline with Tween20 (TBS-T buffer) for 2 h, followed by addition of the primary antibodies against the indicator proteins at 4 °C. Then, the appropriate horseradish peroxidase-coupled secondary antibody was added, and the membrane was washed with TBS-T at room temperature for 2 h. Immune response bands were detected by an enhanced chemiluminescence system and analyzed by Quantity One software (Pierce ECL, western blotting substrate; Thermo Fisher Scientific, Pierce, Rockford, IL, US). ImageJ software [19] was used to quantify each protein band and standardize it to a loading control (GAPDH).

### Cell viability analysis

Cell viability was determined by the Cell Counting Kit-8(CCK8) assay according to the manufacturer’s instructions (China). Cells were used at a density of 2 × 10^3^ cells/well. After treatment with DEX or sterile normal saline, the monolayer was washed three times with PBS, and 10 μL of CCK8 reagent-containing DMEM solution was added and incubated at 37 °C for 2 h. Absorbance was measured using a microplate reader at 450 nm and expressed as percentage of the control value. The cell viability(% of control) was calculated by the following formula [20]: [OD]test/[OD] control X 100.

### Viral constructs and virus production

Full-length cDNA of mouse *Bcat1* (MAGE clone ID 30063465) was cloned into the plvx-puro-3xlag vector. Plvx-puro-3xlag and plko.1-puro-gfp vectors were obtained from Geneline Bioscience. Short hairpin RNA (shRNA) directed against Bcat1 mRNA (shBcat1) was designed and cloned on the plko.1-puro-gfp vector. Target and control sequences were 5′-ccggcccagcatagtatttcgaataccttgtgttttttggttg-3′ and 5′-ccggcccagagaggcggctacttagttg-3′, respectively. Interference of the product generated by employing the primers Bcat1-f (5′-ctcatcacacagcca-3′) and Bcat1-r (5′-ctatccatgtggtcgg-3′) with *Bcat1* expression was verified by PCR. HEK293T cells were transfected with polybrenylamine and Hg transgenic reagent (China) for virus production. The virus-containing supernatant was collected for 48 h and then concentrated by ultracentrifugation at 4500×*g* for 35 min.

### ROS production

ROS production was measured by using a Cellular Reactive Oxygen Species Detection Assay (Abcam, England). Cells were seeded on 20-well plates and incubated with 5 μM dichlorodihydrofluorescein diacetate (DCFH-DA) for 20–30 min and subsequently washed twice with PBS. Changes in relative fluorescence were measured using a fluorescence microscope (Olympus IX51, Japan).

### RNA isolation and quantitative real-time PCR (RT-qPCR)

The gastrocnemius muscle was homogenized in Trizol reagent (Transgen Biotechnology, Beijing, China) on ice, and then separated into organic and aqueous phases by chloroform. After ethanol precipitation, total RNA was extracted from the aqueous phase. RT-qPCR was performed by Transgen Biotechnology (Beijing). *Bcat1* expression was quantified using the thermal cycling dice real-time system and SYBR premix ex Taq II (Takara Bio, Shiga, Japan). All samples were measured in duplicate. GAPDH was used as an internal control to evaluate any variation due to reverse transcription and PCR efficiencies. The forward and reverse primers used for detection of these genes were as follows: mus Gapdh-F: 5′-cgagatgggaagttgtca-3′, Gapdh-R: 5′-cgagatggaagcttgtca-3′; mus Bcat1-F: 5′-ctcatcatcacacaccaccagca-3′, and Bcat1-R: 5′-ctatccatgtggtcgg-3′.

### Whole muscle lysates

The gastrocnemius muscle was homogenized in RIPA buffer (25 mM Tris-HCl, pH 7.6, 150 mM NaCl, 1% NP-40, 1% sodium deoxycholate, and 0.1% SDS) supplemented with a mixture of protease and phosphatase inhibitors (Thermo Fisher Scientific, US). The whole procedure was performed on ice.

### Preparation of frozen sections

Fresh gastrocnemius muscles were dissected, frozen in n-hexane at − 70 °C, and cooled in liquid nitrogen for 2 min. Cross sections (10 μm) were cut at − 20 °C using a cryostat slicer and stained with nicotinamide adenine dinucleotide hydrogen (NADH) according to a previously described method [21].

### Antibodies

Polyclonal anti-BCAT1 antibody (13640–1-ap; Protein Tech) was used for immunofluorescence staining. Western blotting was performed using the following antibodies: rabbit polyclonal anti-BCAT1 (13640–1-ap), anti-pS6K1 (ab59208; Abcam), rabbit monoclonal anti-*p*-mTOR (ab109268; Abcam), anti-mTOR (ab32028; Abcam), anti-S6K1 (ab32359; Abcam), and mouse monoclonal anti-GAPDH (60004–1-lg; Protein Tech).

### Statistical analysis

Data were analyzed using Student’s *t*-test or Mann Whitney U test. Unless specified otherwise, values are presented as mean + standard deviation, and *p* <  0.05 was considered statistically significant. Statistical analyses were performed using GraphPad Prism 6.0 or SPSS 16.0 software.

### Data availability

All other relevant data are available from the corresponding author upon request.

## Results

### Skeletal muscle mass, muscle atrophy, and *Bcat1* expression

**Fig. 1:**
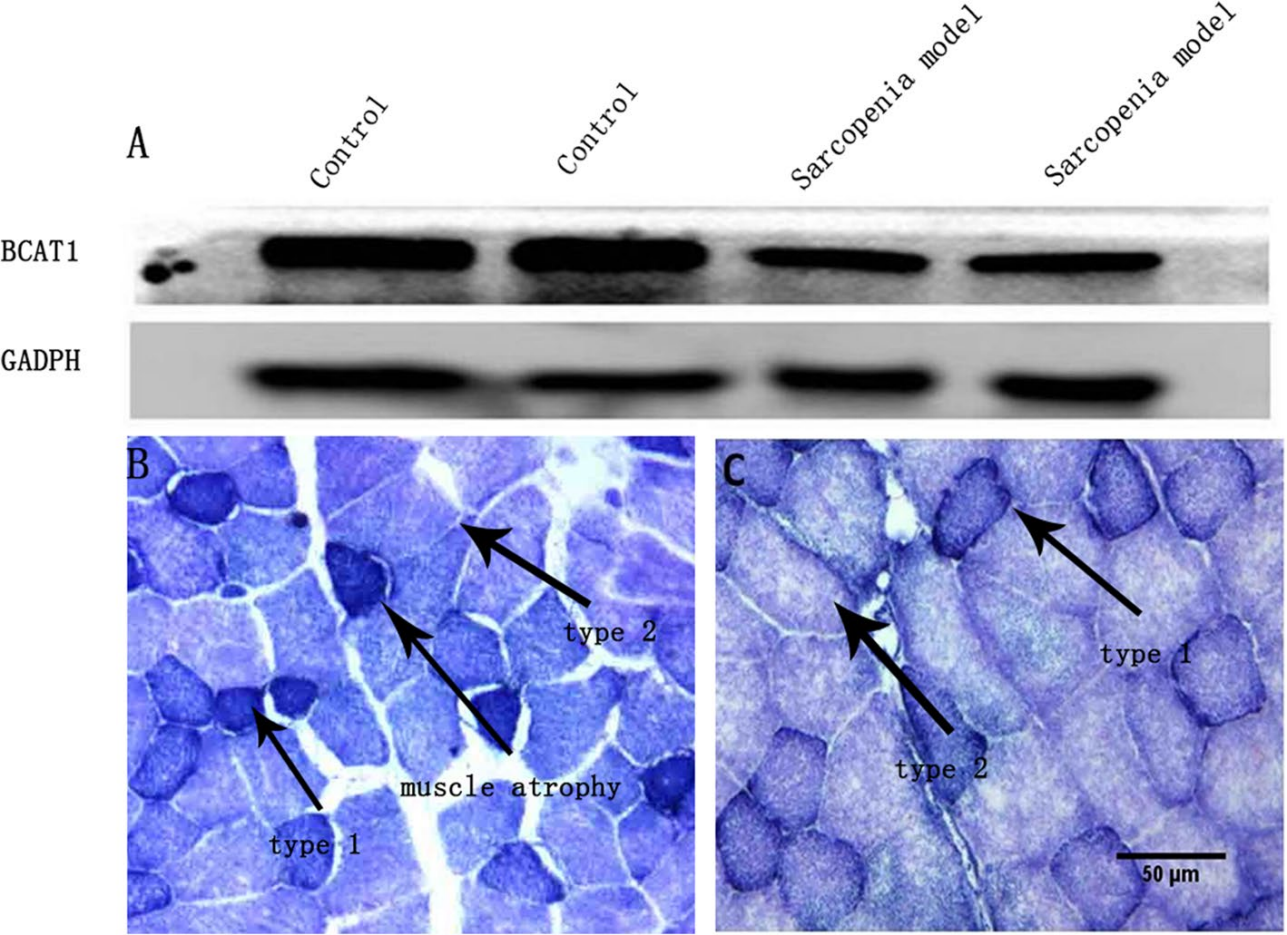
Dexamethasone (DEX) treatment induces muscle atrophy and impaired muscle *Bcat1* expression in mice (*n* = 5). **A** DEX-induced muscle atrophy is associated with reduced *Bcat1* expression. BCAT1 protein expression was determined by western blotting using samples from the gastrocnemius muscle of control mice and mice with muscle atrophy. GAPDH was used as a loading control. **B** NADH staining of the gastrocnemius muscle of mice in the DEX-induced muscle atrophy group. Type 1fibers (dark),type 2 fiber (pale) **C** NADH staining of the gastrocnemius muscle of mice in the control group. Type 1fibers (dark),type 2 fiber (pale)

Animal characteristics are shown in Table 1. The average body weight of DEX-induced sarcopenia mice was lower than that of the mice in the control group. Absolute and total masses of the gastrocnemius and tibialis anterior muscles were measured, and the sarcopenic index was calculated. The absolute muscle mass and sarcopenia index in the sarcopenic group were lower than those in the control group. Compared with the control group, muscle atrophy was more pronounced in the sarcopenia group (Fig. 1B-C).

**Table 1:** Weight of muscles from dexamethasone-induced atrophy in C57BL/6 mice

	Control	DEX	*p* value
Tibialis anterior muscle	0.65 + 0.03	0.46 + 0.03	< 0.01*
Gastrocnemius muscle	1.27 + 0.03	0.96 + 0.08	< 0.01*
Total muscle mass	3.92 + 0.13	2.85 + 0.11	< 0.01*
Body weight	28.75 + 0.54	21.72 + 0.54	< 0.01*
Sarcopenia index	0.136 + 0.003	0.131 + 0.004	0.039*

Data are presented as mean + SEM (*n* = 6); * *p* < 0.05 based on Student’s *t*-test; DEX, dexamethasone

To investigate the role of *Bcat1* in DEX-induced muscle atrophy in mice, we measured *Bcat1* expression level in skeletal muscles (gastrocnemius and tibialis anterior muscles) of mice, and found that *Bcat1* expression in skeletal muscle in the sarcopenic group was lower than that in the control group (Fig. 1A and supplementary material Fig. 1).

### *Bcat1* expression is downregulated in DEX-treated muscle cells

**Fig. 2:**
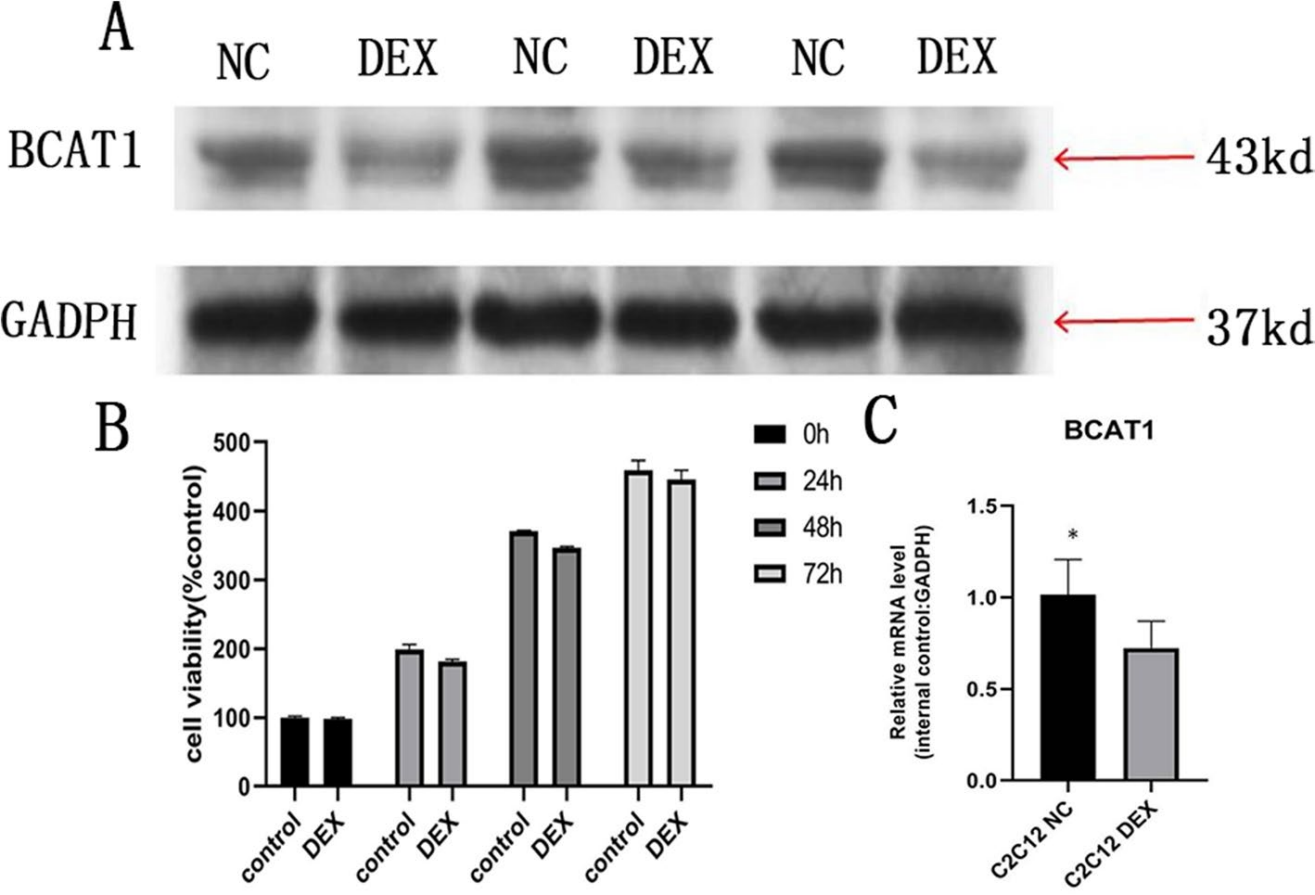
*Bcat1* expression is downregulated in dexamethasone (DEX)-treated C2C12 cells. **A** Immunoblot analysis of BCAT1 protein levels in C2C12 cells cultured in presence and absence of DEX (1 μm, 24 h); *n* = 3 per group. **B** C2C12 cells were cultured in presence of absence of DEX (1 μm, 24 h), after which cell viability was determined by the CCK8 method. Data from three independent experiments are presented as mean + SEM; *n* = 3 per group. **C** *Bcat1* mRNA expression in C2C12 cells with or without DEX treatment (1 μm, 24 h), where relative mRNA levels are representative of the mean value; *n* = 3 per group. ***p < 0.001

To investigate the role of *Bcat1* in muscle atrophy, C2C12 cells were treated with DEX to establish a cellular model of muscle atrophy. We confirmed that C2C12 cell growth was impaired upon DEX treatment (Fig. 2B). Next, we investigated *Bcat1* expression in C2C12 cells. BCAT1 protein expression in growth-restricted C2C12 cells was lower than that in normal muscle cells (Fig. 2 and supplementary material Fig. 2).

### *Bcat1* knockdown impairs growth and promotes ROS production in C2C12 cells

**Fig. 3:**
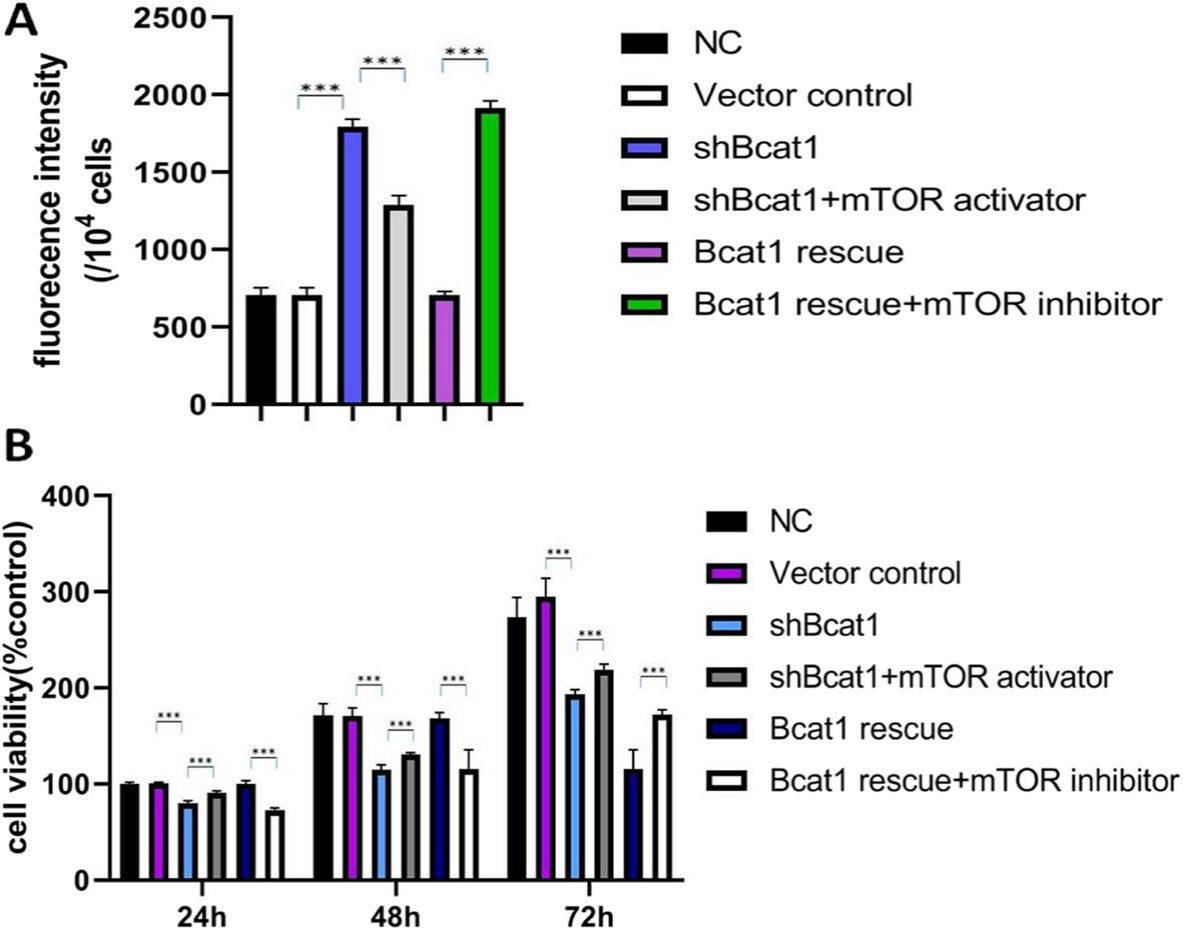
*Bcat1* knockdown impairs cell growth and promotes ROS production in muscle cells. **A** ROS concentration in C2C12 cells was detected by the fluorescence method. Grouping: control vector (shCtrl), sh*Bcat1*, sh*Bcat1* + mTOR activator, sh*Bcat1* + shRNA-resistant *Bcat1* cDNA (rescue), sh*Bcat1* + shRNA-resistant *Bcat1* cDNA (rescue) + mTOR inhibitor, n = 3, cells were analyzed at 24 h post-treatment. **B** Viability of C2C12 cells was detected by the CCK8 method. Grouping: control vector (shCtrl), sh*Bcat1*, sh*Bcat1* + mTOR activator, sh*Bcat1* + shRNA-resistant *Bcat1* cDNA (rescue), sh*Bcat1* + shRNA-resistant *Bcat1* cDNA (rescue) + mTOR inhibitor, *n* = 3, cells were analyzed at 24 h, 48 h, 72 h post-treatment. Data from three independent experiments are presented as mean + SEM. ***p < 0.001

Since *Bcat1* expression was downregulated in a DEX-induced cellular muscle atrophy model, we wanted to determine the role of *Bcat1* in the pathogenesis of muscle atrophy. Therefore, we prepared lentivirus, which could knock down the expression of *Bcat1*, the first enzyme in the BCAA catabolic pathway, in C2C12 muscle cells. *Bcat1* knockdown inhibited the growth rate of C2C12 cells from day 2 (Fig. 3B). Next, we studied the mechanisms by which *Bcat1* contributed to muscle cell growth. Therefore, we analyzed whether loss of *Bcat1* increased ROS production. Compared with the vector control group, shBcat1 (*Bcat1* gene knockdown) inhibited the growth of muscle cells in vitro. Further evidence suggested that *Bcat1* knockdown not only inhibited the growth of the muscle cells but also increased the production of ROS (Fig. 3A), which in turn inhibited cell growth. Quantitative analysis of cell viability according to the CCK8 assay showed that cell growth in the *Bcat1* knockdown group was decreased after 24 h, which was not statistically significant. After 48 h, quantitative data showed that cell growth had decreased significantly in response to *Bcat1* knockdown compared with the control group (Fig. 3B). *Bcat1* knockdown also significantly promoted ROS production (Fig. 3A). Combined introduction of anti-shRNA *Bcat1* cDNA or the mTORC1 stimulator MYH1485 alleviated both impaired cell growth and increased ROS production (Fig. 3A-B).

### mTOR contributes to *Bcat1* function in muscle cells

**Fig. 4:**
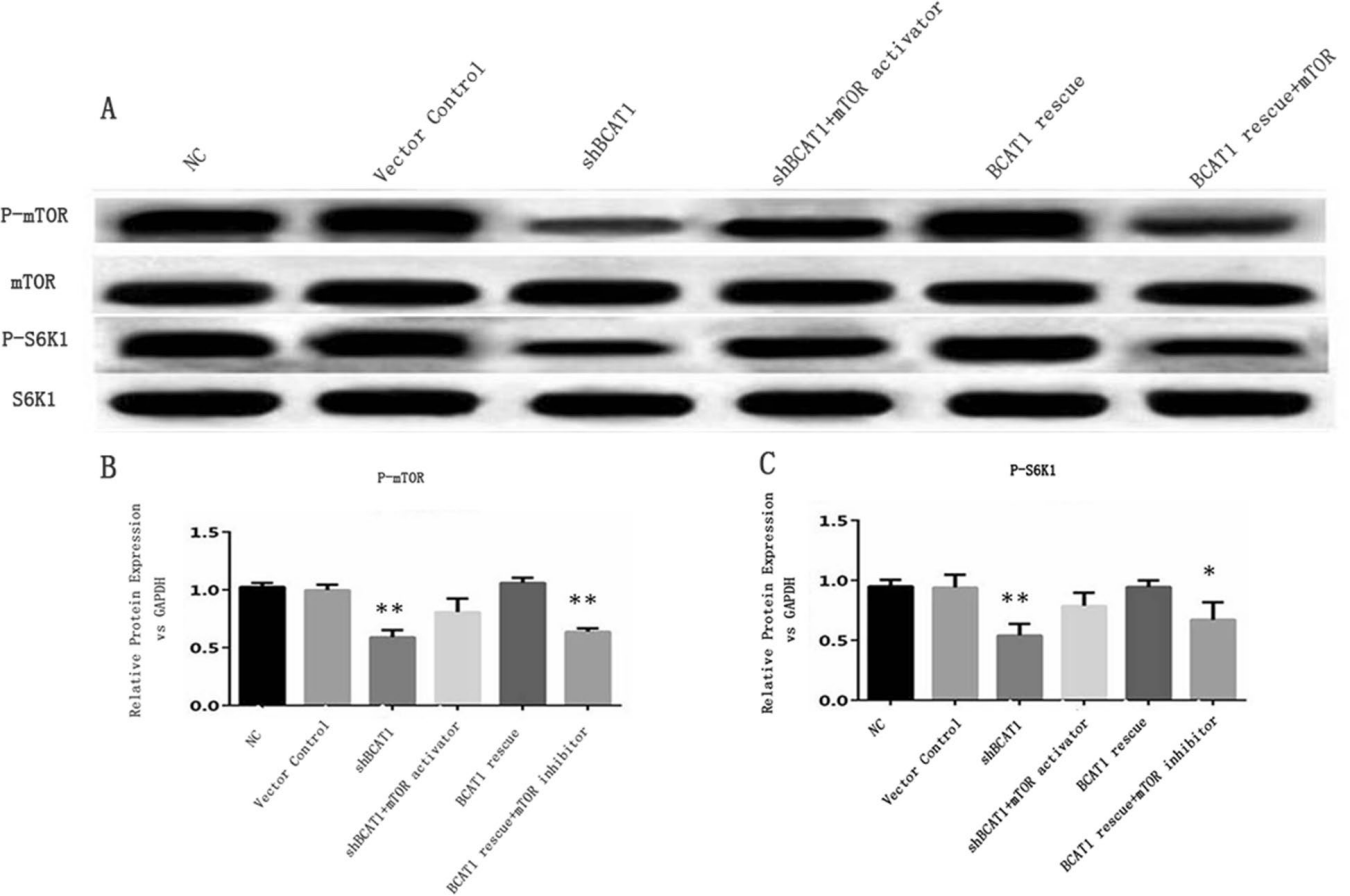
Inhibitory effect of Bcat1 knockdown on activation of the mTORC1-S6K1 signal pathway. **A** Immunoblot analysis of mTOR, *p*-mTOR, *p*S6K1, and S6K1 in mouse C2C12 cells. Grouping: control vector (shCtrl), sh*Bcat1*, sh*Bcat1* + mTOR activator, sh*Bcat1* + shRNA-resistant *Bcat1* cDNA (rescue), sh*Bcat1* + shRNA-resistant *Bcat1* cDNA (rescue) + mTOR inhibitor, *n* = 3, cells were analyzed at 24 h post-treatment. Representative images of protein levels of mTOR, p-mTOR, S6K1, and pS6K1 in muscle cells are presented. **B**-**C** Quantitative analysis of protein levels of *p*-mTOR and *p*S6K1, where the relative band intensity was normalized to GAPDH. Data from three independent experiments are presented as mean + SEM. *p < 0.05, **p < 0.01

Finally, we asked how the metabolic regulator *Bcat1* affected muscle cell growth and ROS production. To understand this, we analyzed ROS levels and activation of the mTORC1/S6K1 pathway in myocytes. Western blotting was used to determine the phosphorylation status and total protein concentration of the mTORC1 downstream target S6K1 in the gastrocnemius muscle. As shown in Fig. 4 and supplementary material Fig. 4, the ratios of *p*-mTOR and mTOR, as well as those of *p*S6K1 and S6K1 in the *Bcat1* knockdown group were significantly reduced compared with the control group. To further confirm the role of the mTOR1/S6K1 axis in *Bcat1* knockdown-induced muscle cell growth impairment, we used shRNA-resistant *Bcat1* cDNA and mTOR activators/inhibitors. Compared with the shBcat1 plasmid, the viability of muscle cells transfected with anti-Bcat1 shRNA could be recovered significantly. In addition, myocytes treated with the mTOR activator MYH1485 showed improved cell viability, while muscle cells treated with an mTOR inhibitor were damaged. Treatment with MYH1485, an activator of mTOR, was associated with significantly reduced cell viability after shBcat1 transfection. Rapamycin, an mTOR inhibitor, blocked the effect of *Bcat1* on cell viability and growth. In conclusion, compared with NT myocytes, the phosphorylation levels of mTORC1 and S6K1 in growth-impaired myocytes were lower.

**Figure:**
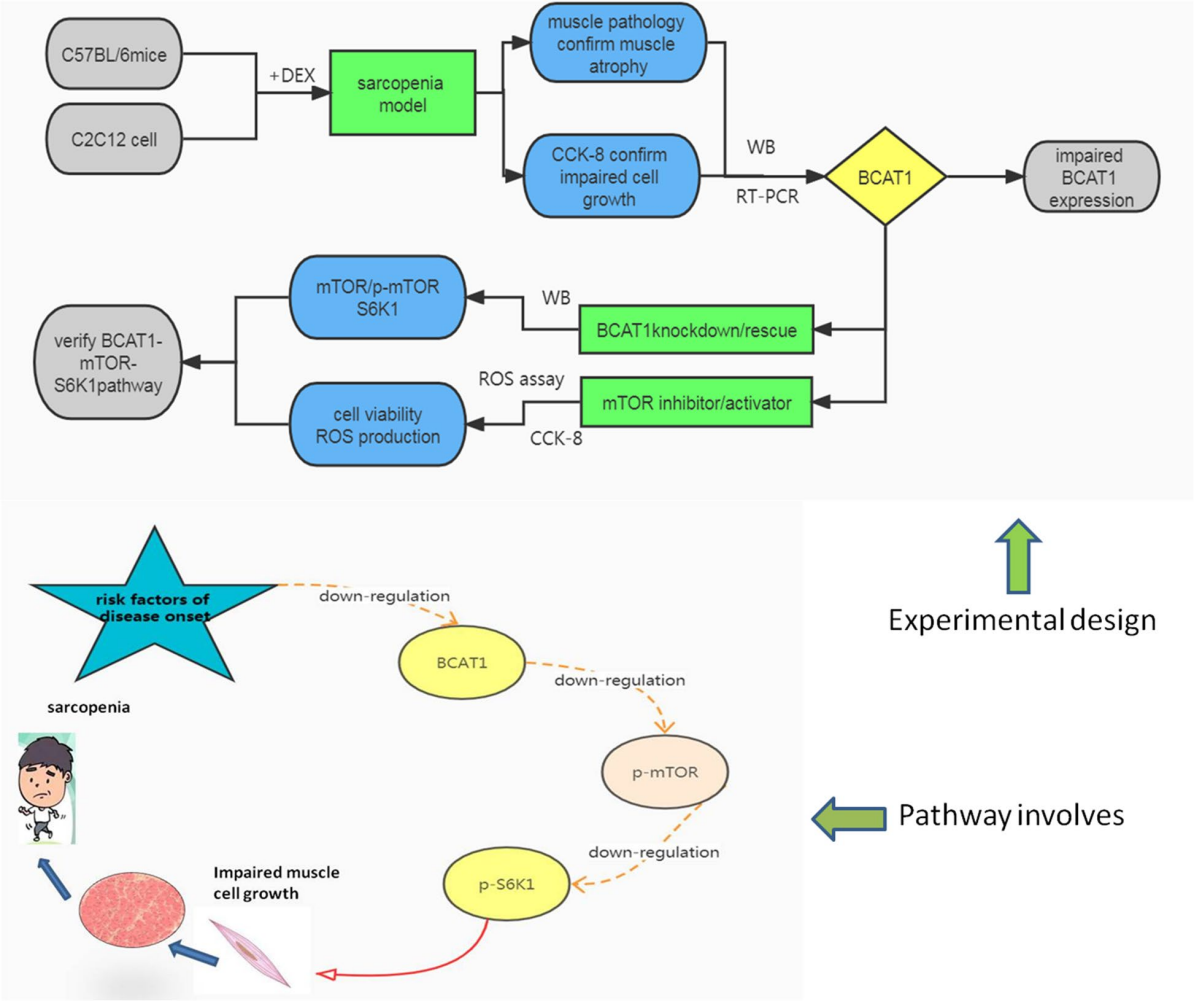
Diagam of experimental design and tested pathway

## Discussion

Although *BCAT1* is expressed in skeletal muscle and plays an important role in muscle energy metabolism [22, 23], the molecular basis of its involvement in the pathogenesis of muscle atrophy is still unclear.

In this study, we put forward some important suggestions. First, we found that *Bcat1* expression was downregulated in a model of muscle atrophy. Second, we demonstrated for the first time that *Bcat1* could maintain muscle cell growth and reduce ROS production. Third, we found that *Bcat1* regulated muscle cell growth and ROS production through the mTORC1/S6K1 signaling pathway. These results suggest that downregulation of *Bcat1* and mTORC1/S6K1 pathway is one of the reasons for impaired cell growth in atrophic muscle cells.

BCAAs are the cornerstone of protein synthesis and energy provision [24]. However, the relative importance of the bioenergy contribution of amino acids in muscle is still unclear under both physiological and pathological conditions. *BCAT1* is the first enzyme to initiate the catabolism of BCAAs. Our study provides additional evidence that downregulation of *BCAT1* had adverse effects on muscle cell growth and ROS production.

Accumulating evidence suggests that regulation of mTOR is essential for cell growth. Cardiomyocyte-specific mTOR depletion led to decreased proliferation and increased apoptosis, resulting in a decrease in embryonic heart size [21]. However, whether *BCAT1* affected the regulation of mTOR1/S6K1 signal transduction remained unclear. This study provides an insight into the roles of the BCAT1/mTOR/S6K1 pathway. Our results indicate for the first time that *BCAT1* depletion significantly reduced the activation of the mTORC1/S6K1 signaling pathway in muscle cells. Therefore, we established a link between *BCAT1* and muscular atrophy/sarcopenia.

*BCAT1* increases the mitochondrial content by activating key regulatory factors of mitochondrial biogenesis [25]. Our results suggest that *BCAT1* may promote mitochondrial function and cell growth by reducing ROS production and regulating a variety of nutritional sensors, including mTOR and S6K1. Interestingly, we found that *BCAT1* could activate mTOR signaling and downregulate ROS production. Rapamycin inhibited mTOR signaling and blocked the effect of *BCAT1* on ROS production and subsequent cell growth impairment. Because *BCAT1* depletion decreased the levels of *p*-mTOR and pS6K1, *BCAT1* expression is presumed to positively regulate mTORC1 activation. Importantly, shRNA-resistant *BCAT1* cDNA and mTOR activators effectively reversed the growth impairment induced by *BCAT1* knockdown and led to increased *p*S6K1 levels in a rapamycin-sensitive manner. In conclusion, we assigned an important role to the BCAT1/mTOR axis with regard to the pathogenesis of muscle atrophy, and provide theoretical proof for reducing muscle atrophy by regulating the BCAT1/mTOR pathway. However, further studies are needed to explain how the catabolic enzyme BCAT1 activates mTOR in muscle cells.

In conclusion, we demonstrated that *BCAT1* is essential for the growth of muscle cells. *BCAT1* maintains muscle cell growth by activating mTOR signaling and reducing ROS production. Therefore, stable *BCAT1* expression is necessary to maintain the growth of muscle cells and reduce ROS production. Moreover, *BCAT1* may be a potential target for the treatment of muscle atrophy. Our observations highlight potential therapeutic targets for nutritional or pharmaceutical interventions aimed at reducing severe sarcopenia in patients with muscular atrophy. In view of the limitations faced in the current study, we have no evidence that *BCAT1* regulates the mTORC1/S6K1 signaling pathway in vivo. Moreover, we have not explored other important factors or molecules related to muscle atrophy, such as inflammation and Notch signaling, among others. To to compensate for this, we plan to use the *BCAT1* knockdown animal model to verify our findings and explore other effects and mechanisms of *BCAT1* in muscle atrophy. Accordingly, further experiments are needed to confirm whether *BCAT1* is also involved in the development of muscle atrophy in vivo, and whether changes in BCAT1 levels are associated with the progression of muscle atrophy. Finally, the results have not been verified in sarcopenia patients. We will try to use human tissues and conducting clinical research when conditions permit in the future. In all, the BCAA catabolic enzyme BCAT1 is essential for the growth of muscle cells. *BCAT1* expression contributes to sustained growth of muscle cells by activating mTOR signaling and reducing ROS production.

## Supplementary Information

**Additional file 1.**

**Additional file 2.**

**Additional file 3.**

## Data Availability

Authors declare that data and materials described in the manuscript are freely available to any scientist wishing to use them, without breaching participant confidentiality. The contact should be made via the corresponding author happyluckyfish@163.com

## Abbreviations

BCAAs: Branched-chain amino acids
BCKAs: Branched-chain keto acids
BCAT1: Branched-chain aminotransferase 1
WT: Wild-type
mTOR: Mammalian target of rapamycin
mTORC1: Complex 1 of mTOR pathway
α-KG: α-ketoglutarate
S6K1: 70-kDa ribosomal protein S6 kinase 1
