# Supplementary figures and images for: Impaired expression of BCAT1 relates to muscle atrophy of mouse model of sarcopenia
Source: BMC Musculoskelet Disord. 2022 May 13;23:450. doi: 10.1186/s12891-022-05332-7 (PMC9102634; doi:10.1186/s12891-022-05332-7)

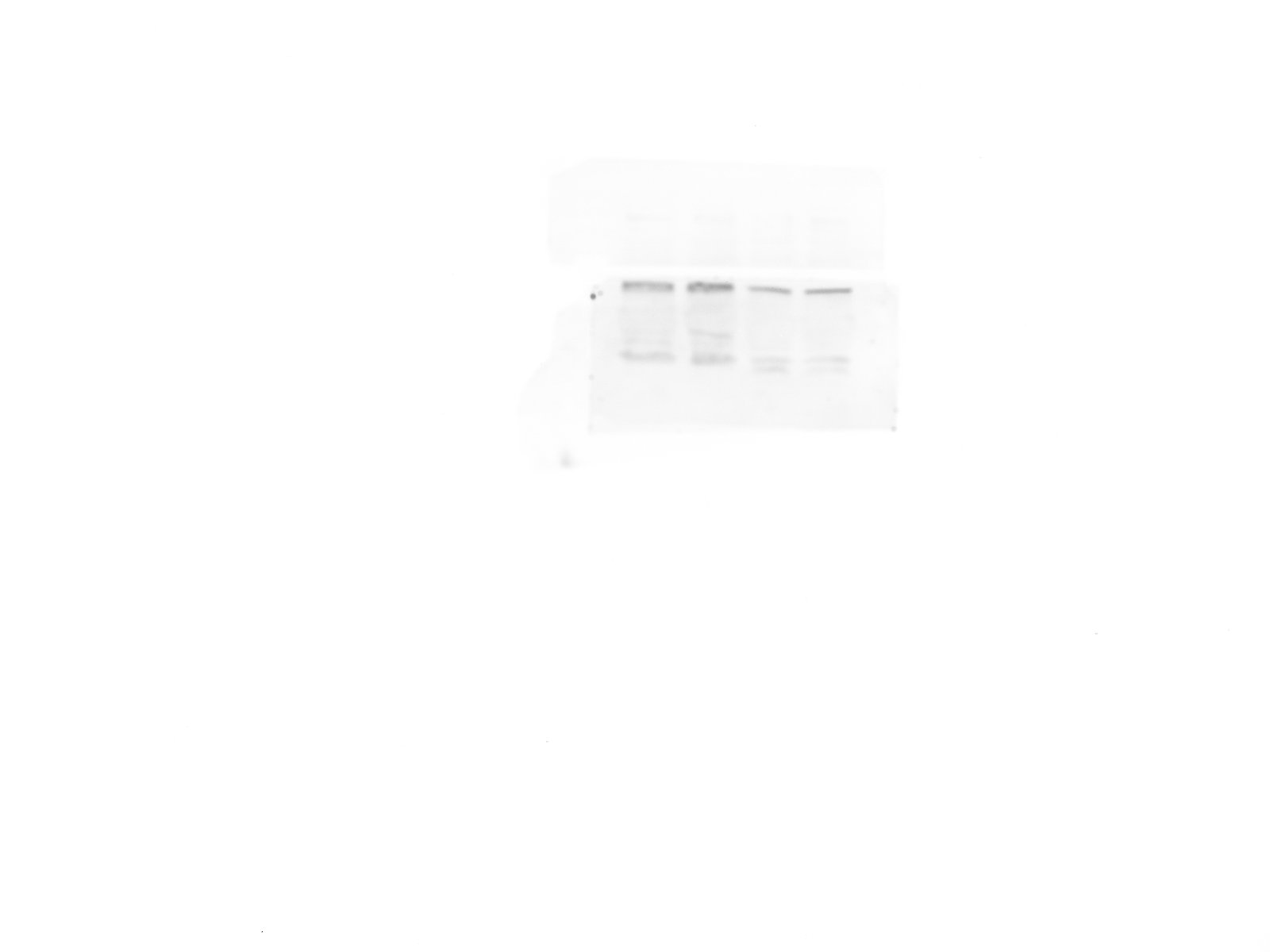
’

BCAT1-exposure1


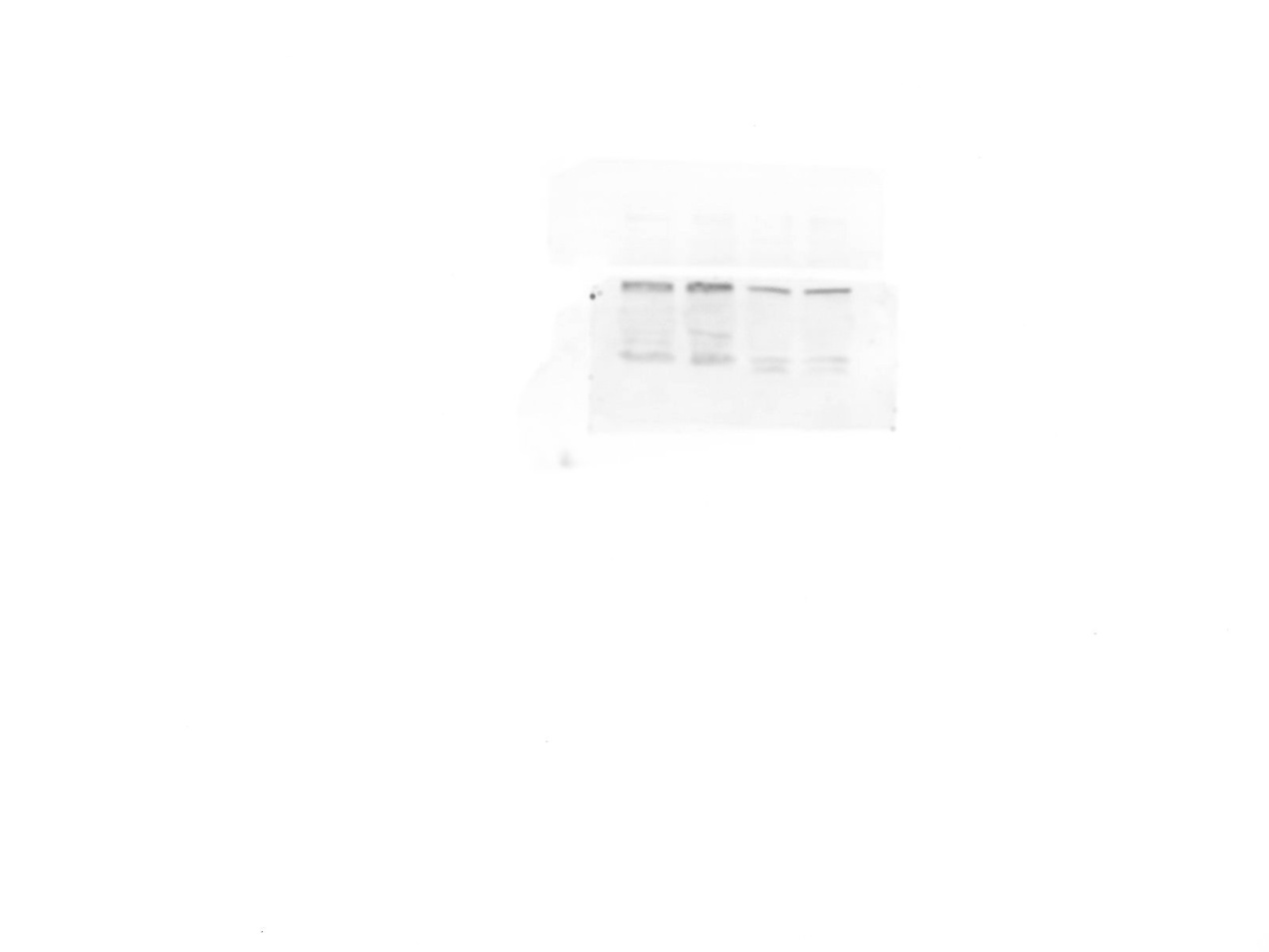


BCAT1-exposure2


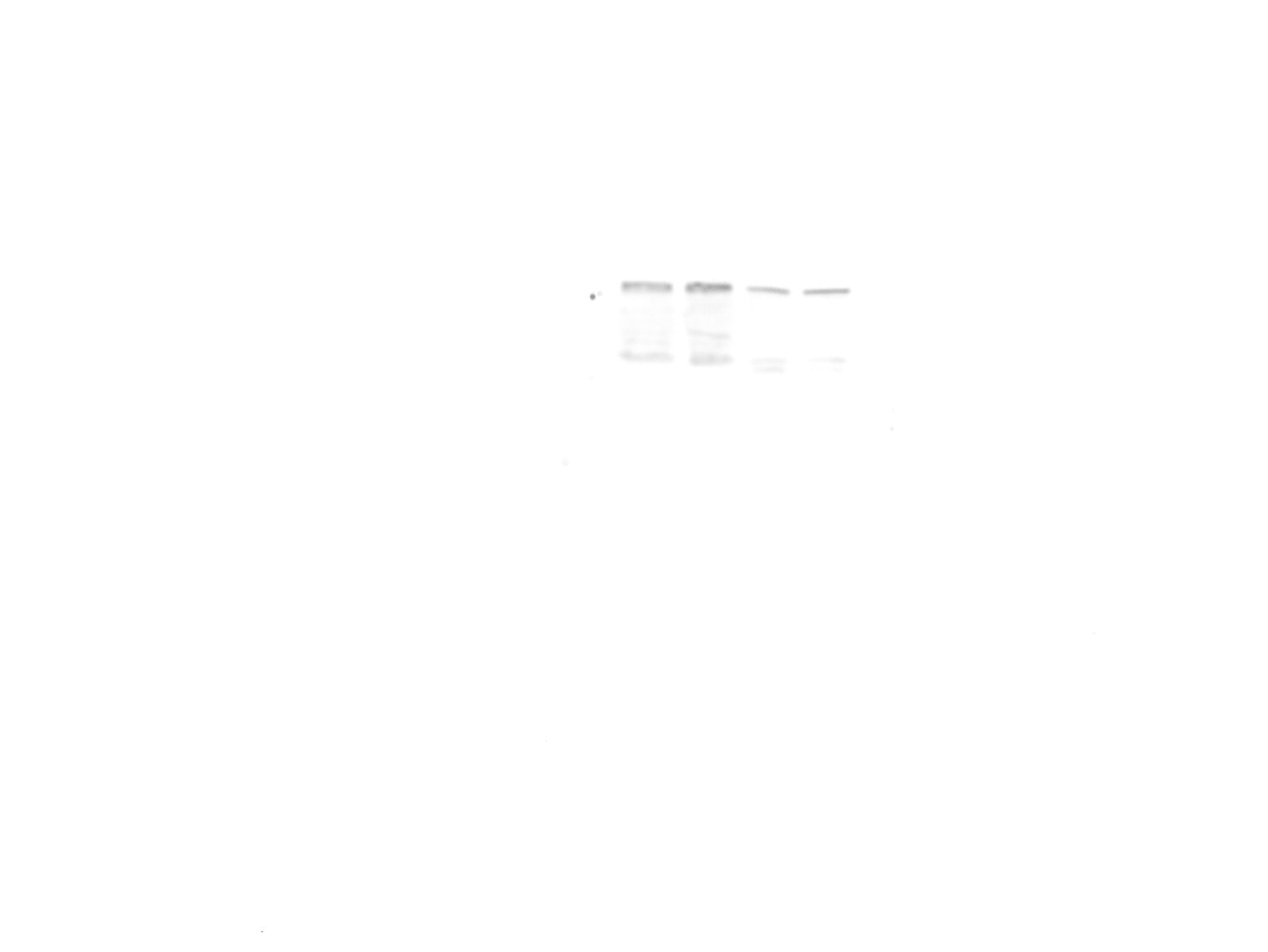


BCAT1-exposure3


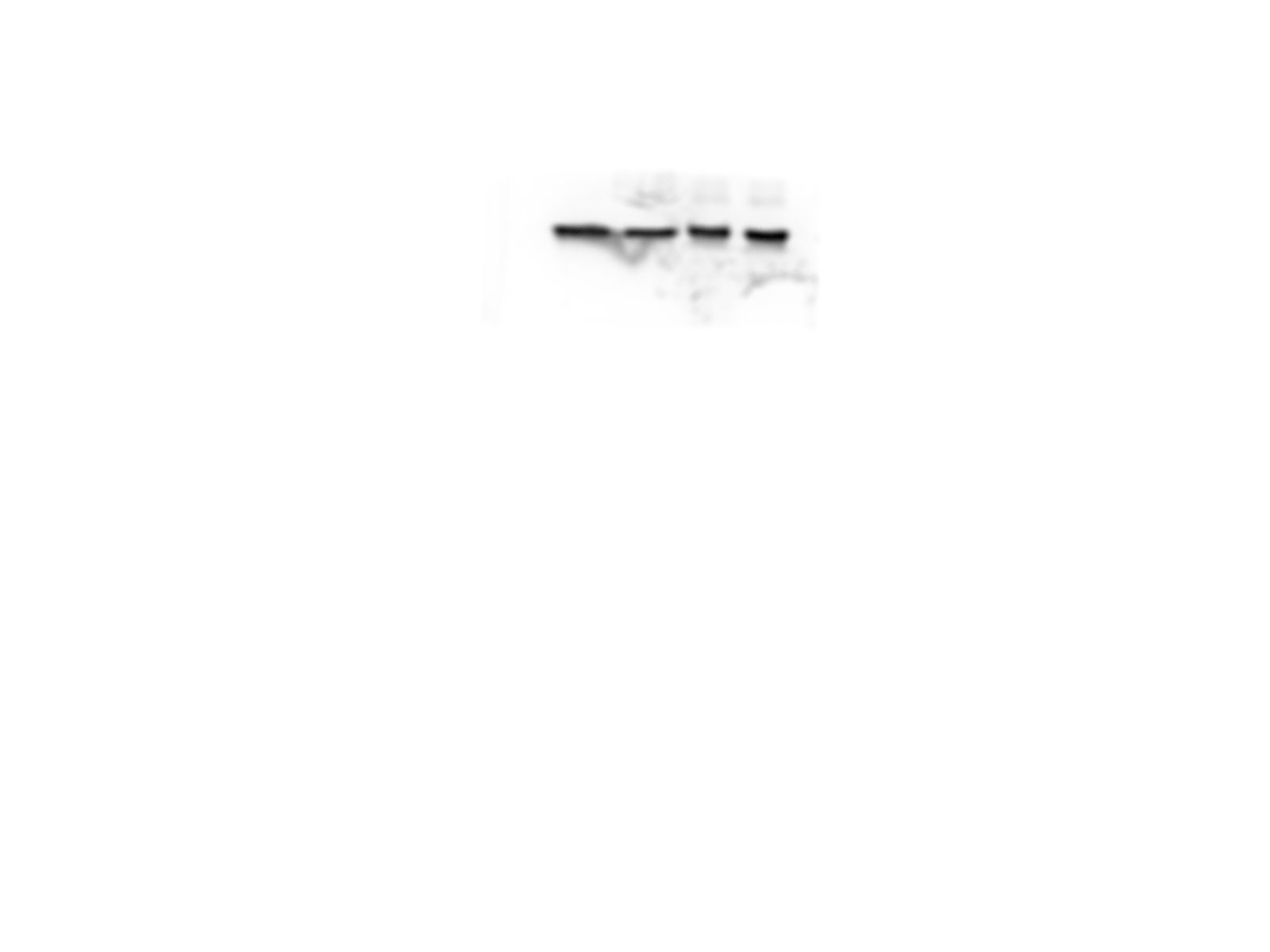


GADPH-exposure1


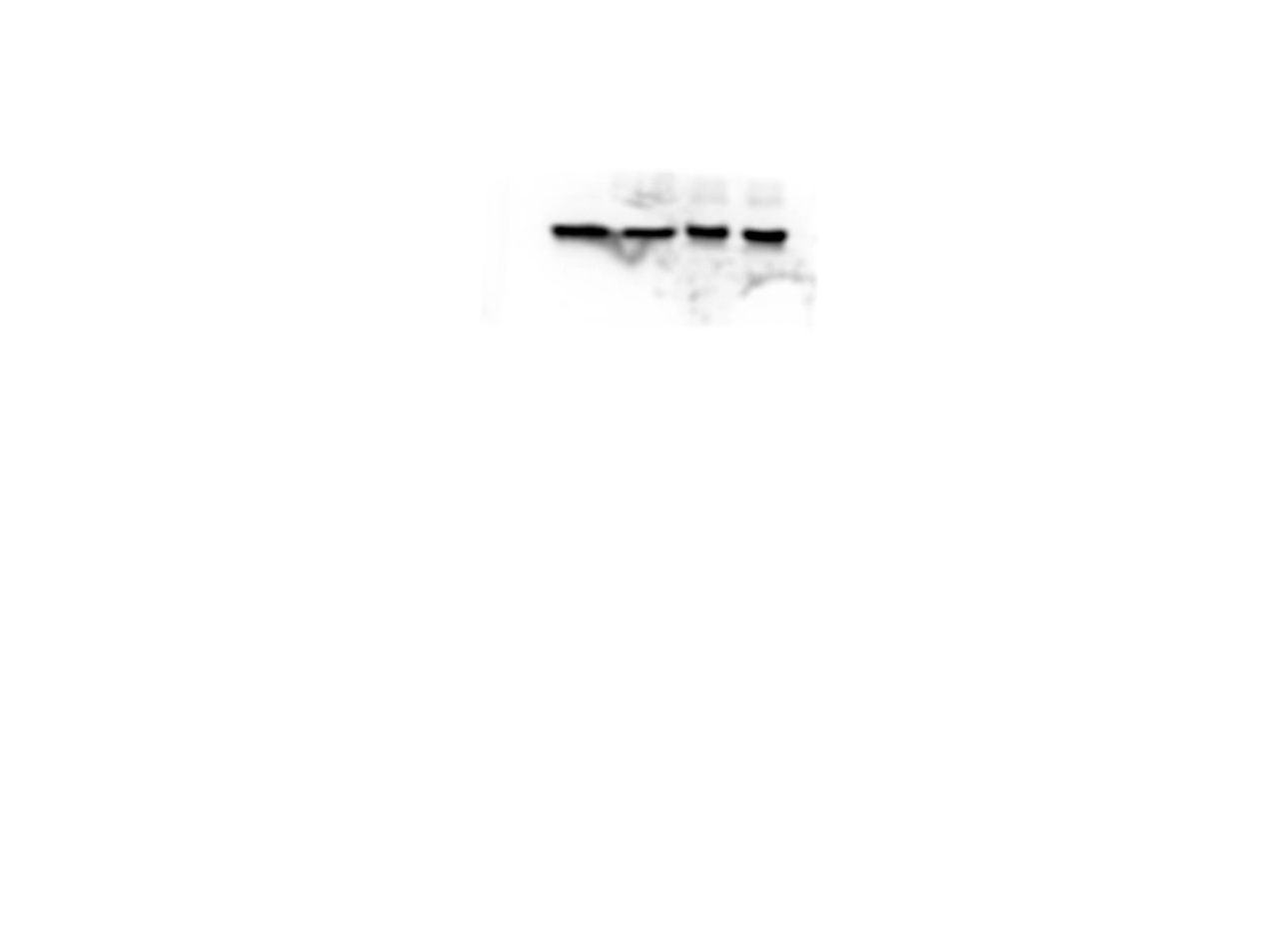


GADPH-exposure2


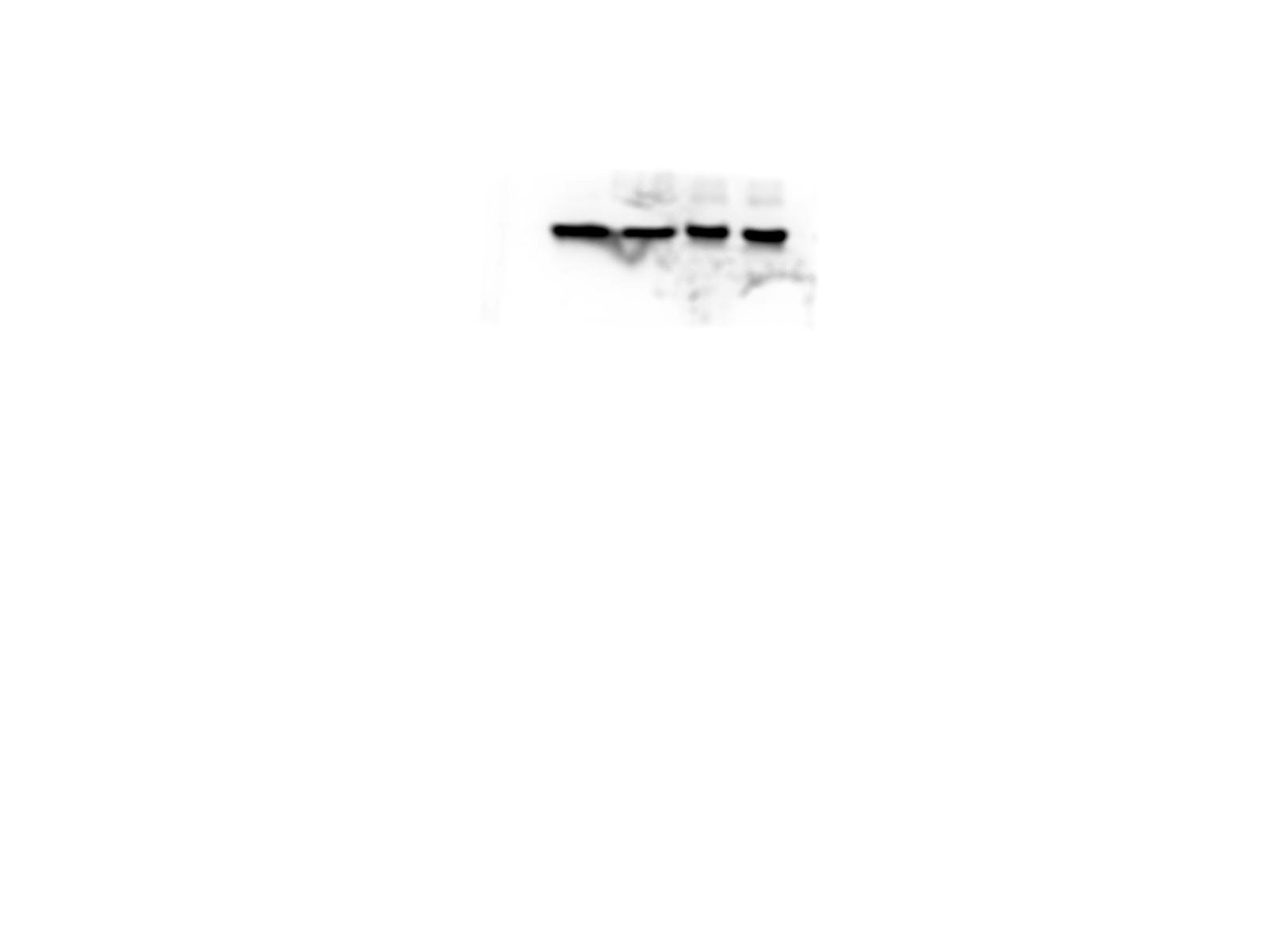


GADPH-exposure3

Supplement: Supplementary file 1 — Additional file 1. [file 12891_2022_5332_MOESM1_ESM.docx]

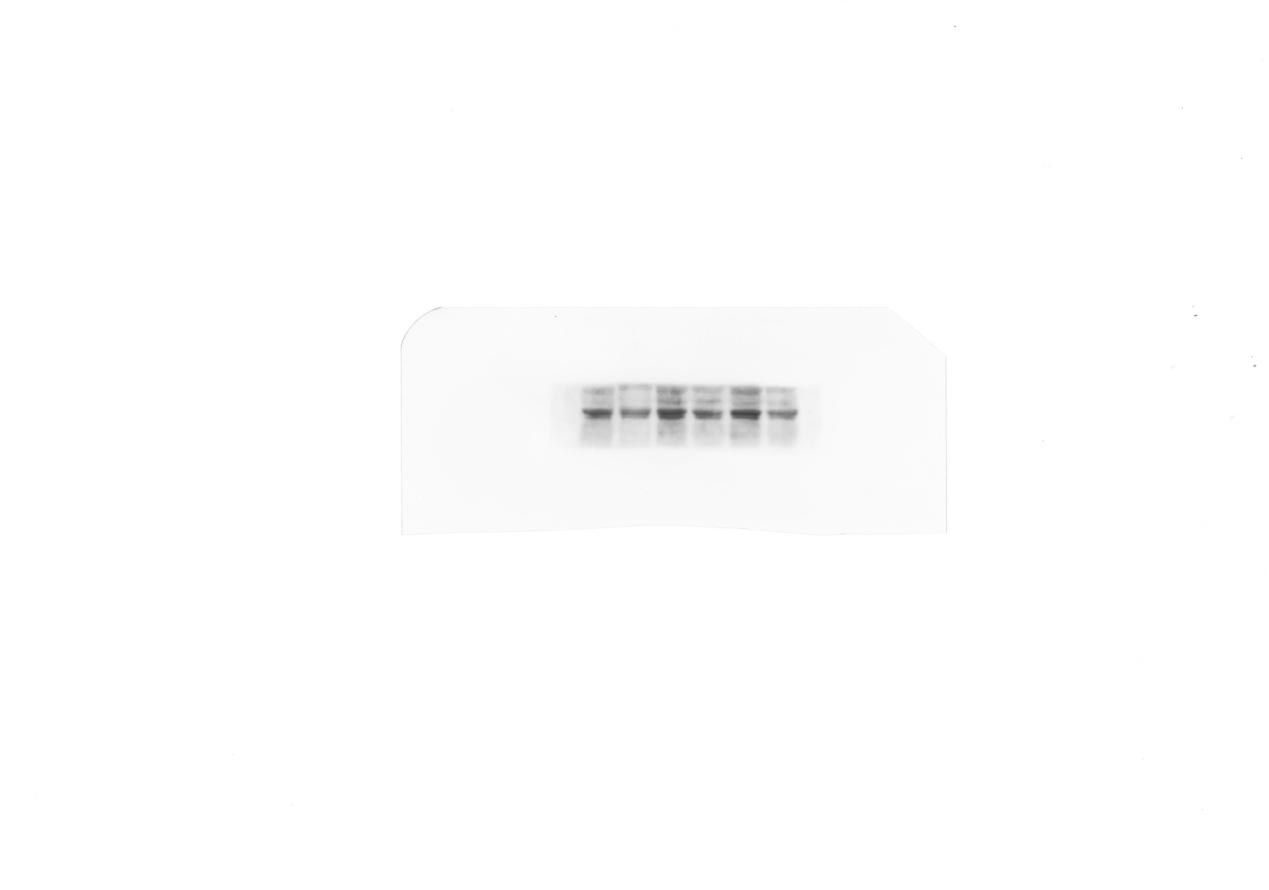


BCAT1-exposure1


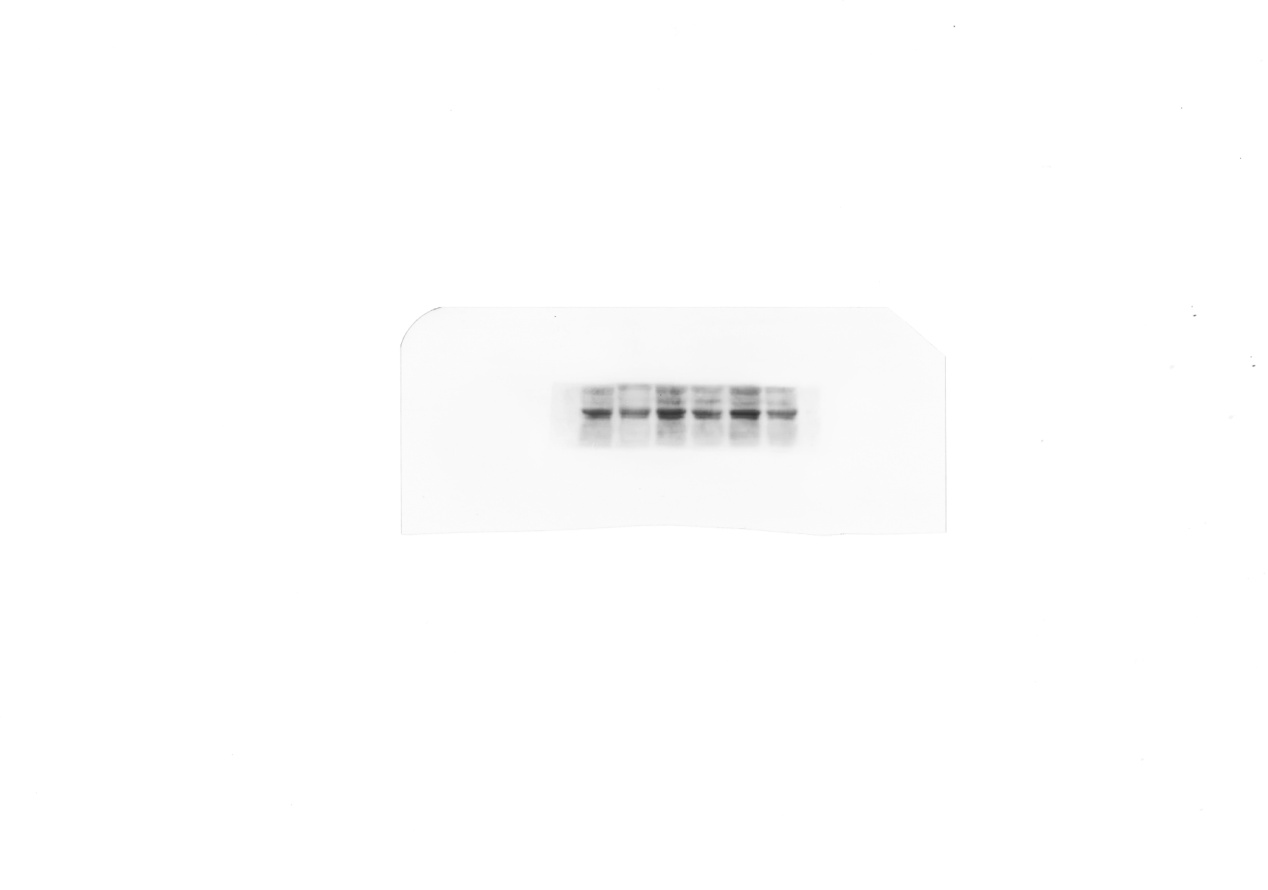


BCAT1-exposure2


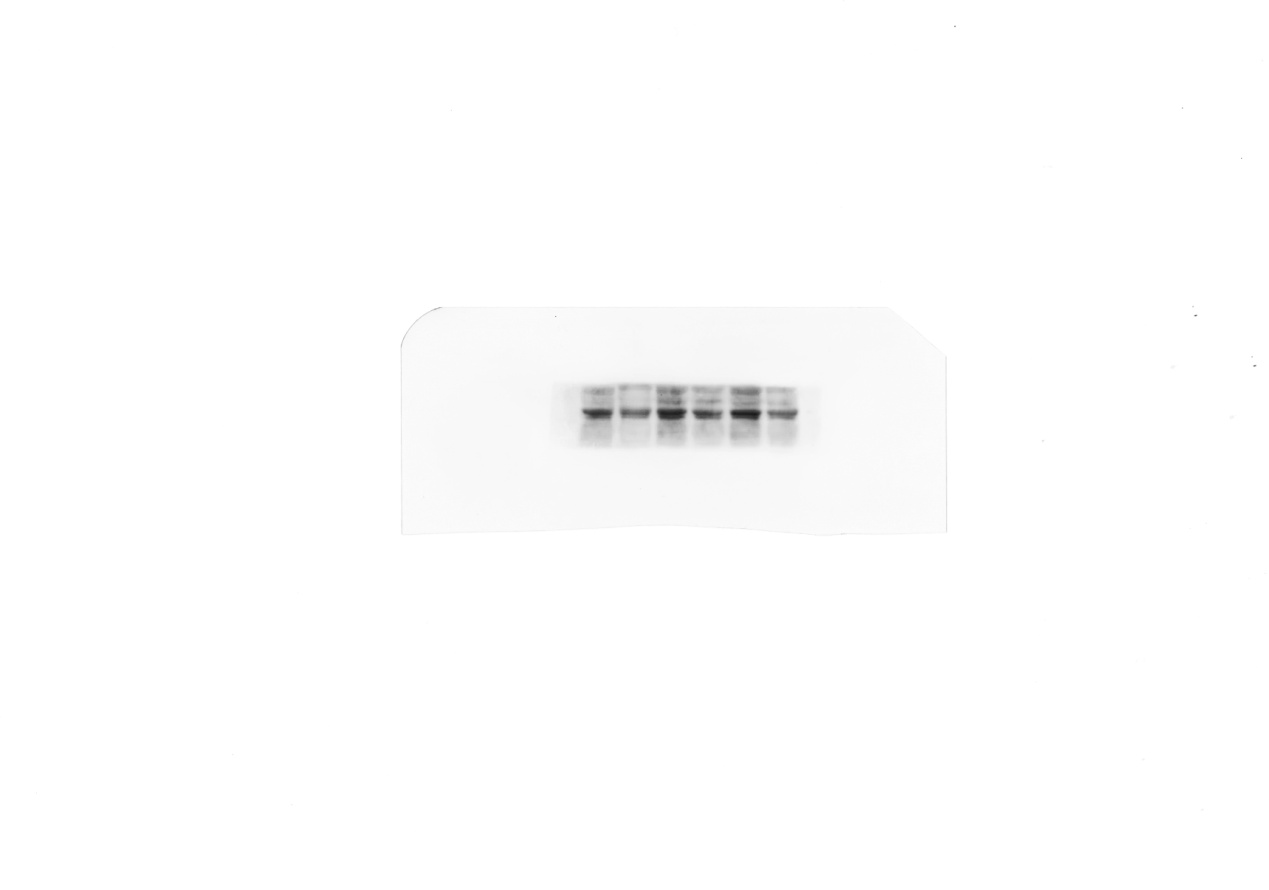


BCAT1-exposure2


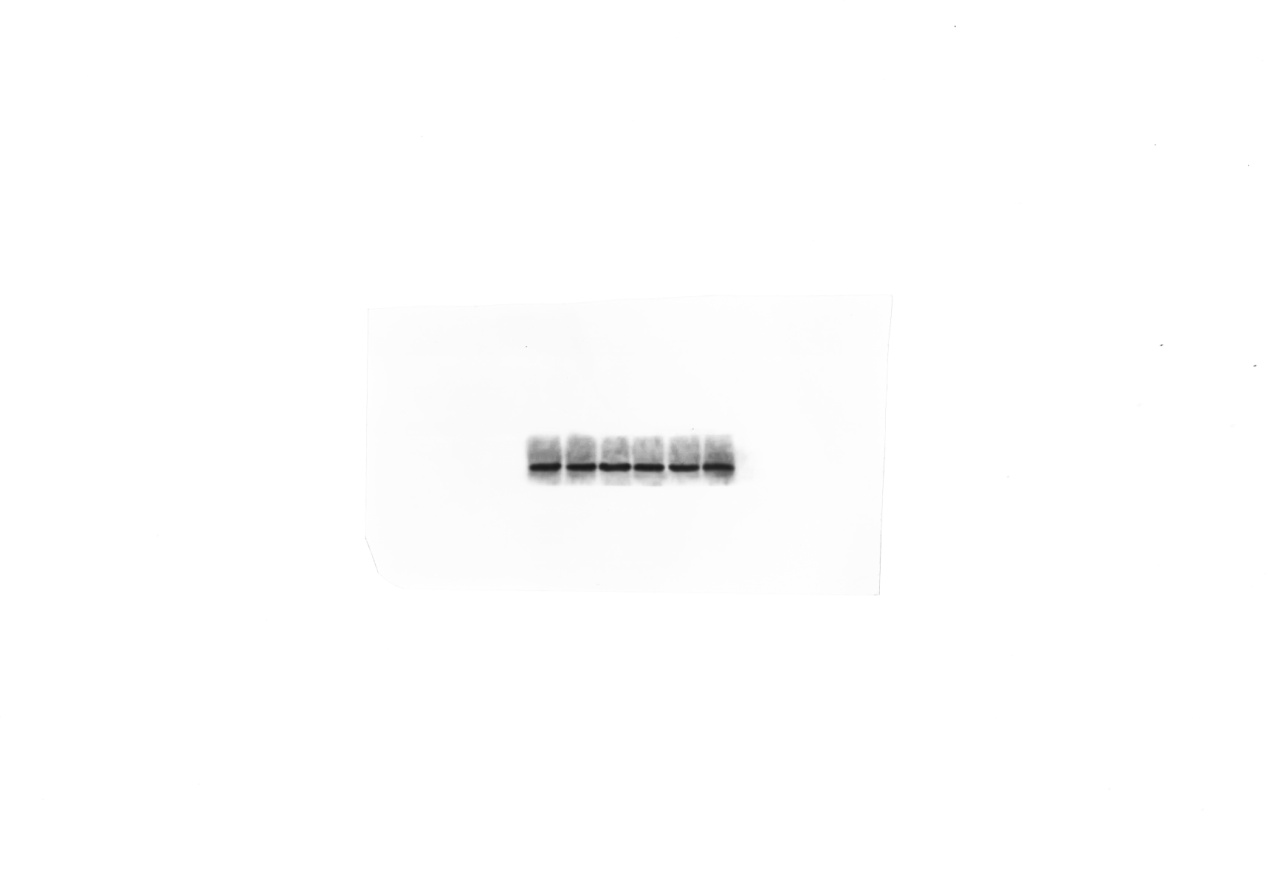


GADPH-exposure1


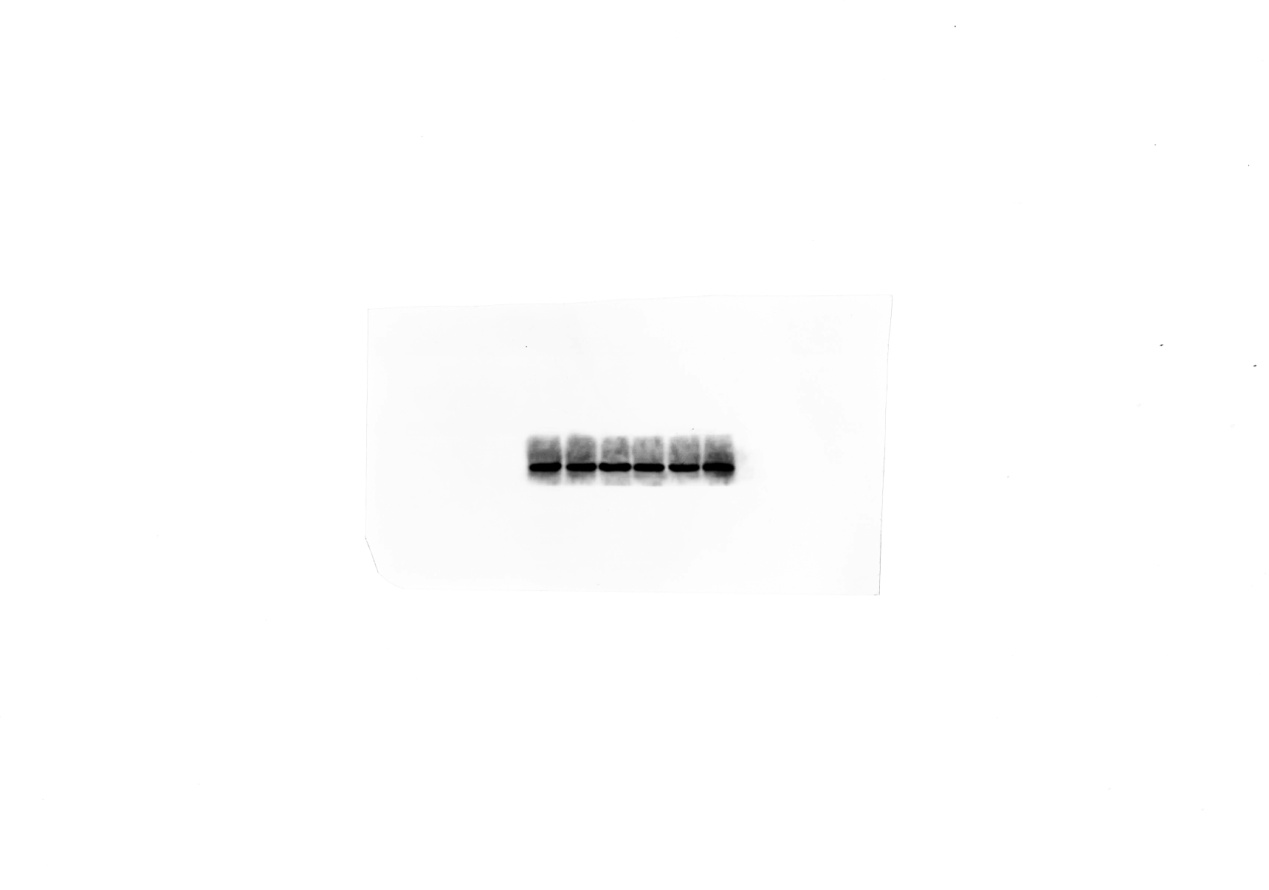


GADPH-exposure2


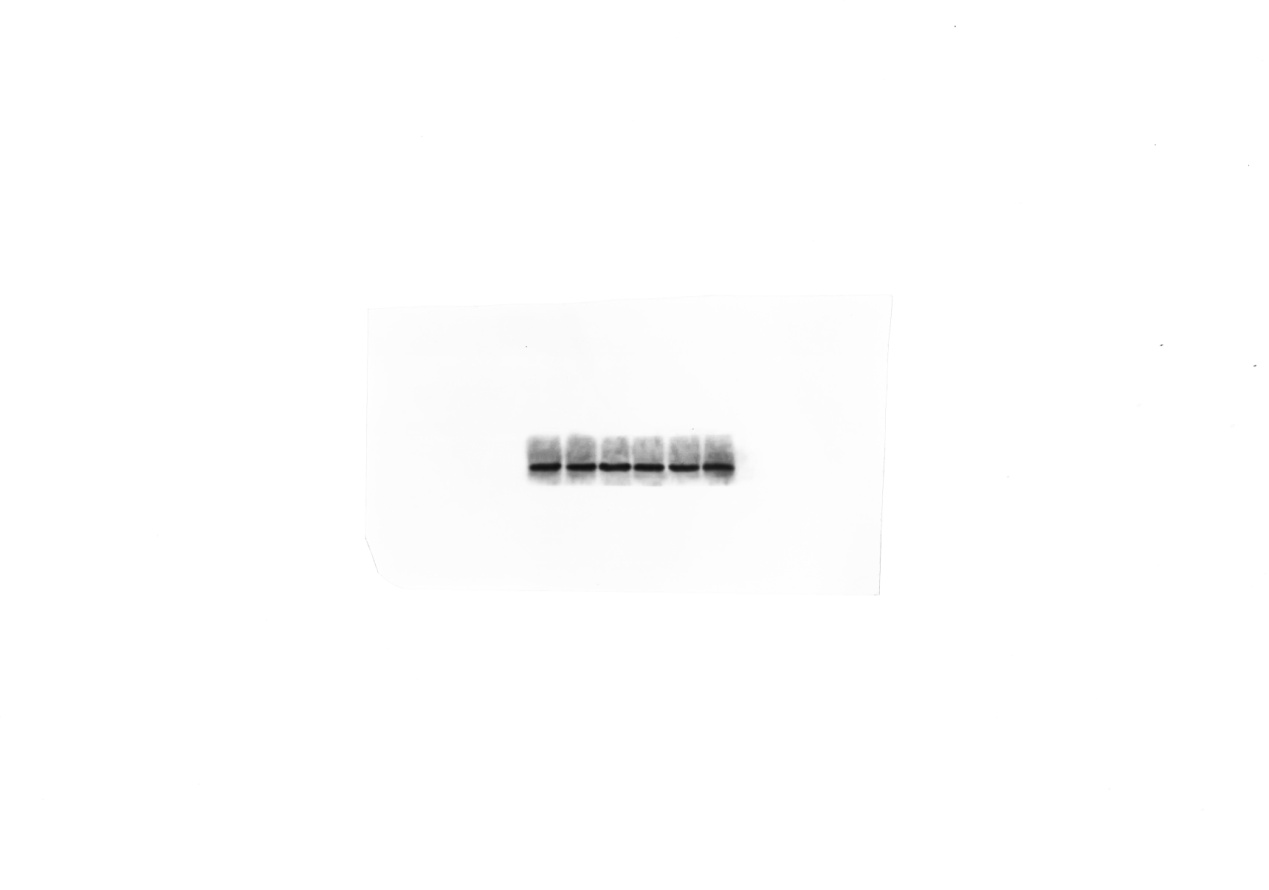


GADPH-exposure3

Supplement: Supplementary file 2 — Additional file 2. [file 12891_2022_5332_MOESM2_ESM.docx]
