# Supplementary material for: Impaired expression of BCAT1 relates to muscle atrophy of mouse model of sarcopenia
Source: BMC Musculoskelet Disord. 2022 May 13;23:450. doi: 10.1186/s12891-022-05332-7 (PMC9102634; doi:10.1186/s12891-022-05332-7)
Supplement: Supplementary file 3 — Additional file 3. [file 12891_2022_5332_MOESM3_ESM.docx]

GADPH





HIF1a





mTOR





p-mTOR





p-S6K1





S6K1
